# Low Dose PET Image Reconstruction with Total Variation Using Alternating Direction Method

**DOI:** 10.1371/journal.pone.0166871

**Published:** 2016-12-22

**Authors:** Xingjian Yu, Chenye Wang, Hongjie Hu, Huafeng Liu

**Affiliations:** 1 State Key Laboratory of Modern Optical Instrumentation, Department of Optical Engineering, Zhejiang University, Hangzhou, China; 2 Sir Run Run Shaw Hospital, Zhejiang University, Hangzhou, China; Chongqing University, CHINA

## Abstract

In this paper, a total variation (TV) minimization strategy is proposed to overcome the problem of sparse spatial resolution and large amounts of noise in low dose positron emission tomography (PET) imaging reconstruction. Two types of objective function were established based on two statistical models of measured PET data, least-square (LS) TV for the Gaussian distribution and Poisson-TV for the Poisson distribution. To efficiently obtain high quality reconstructed images, the alternating direction method (ADM) is used to solve these objective functions. As compared with the iterative shrinkage/thresholding (IST) based algorithms, the proposed ADM can make full use of the TV constraint and its convergence rate is faster. The performance of the proposed approach is validated through comparisons with the expectation-maximization (EM) method using synthetic and experimental biological data. In the comparisons, the results of both LS-TV and Poisson-TV are taken into consideration to find which models are more suitable for PET imaging, in particular low-dose PET. To evaluate the results quantitatively, we computed bias, variance, and the contrast recovery coefficient (CRC) and drew profiles of the reconstructed images produced by the different methods. The results show that both Poisson-TV and LS-TV can provide a high visual quality at a low dose level. The bias and variance of the proposed LS-TV and Poisson-TV methods are 20% to 74% less at all counting levels than those of the EM method. Poisson-TV gives the best performance in terms of high-accuracy reconstruction with the lowest bias and variance as compared to the ground truth (14.3% less bias and 21.9% less variance). In contrast, LS-TV gives the best performance in terms of the high contrast of the reconstruction with the highest CRC.

## Introduction

Positron emission tomography (PET) is a nuclear image modality that can produce 3D functional images of biological processes inside the human body [1]. PET has now become an indispensable tool in cardiac/brain research and cancer diagnosis/treatment. However, the reconstruction of low-dose PET images has remained a challenge because of the large amount of noise and the sparse spatial resolution [2]. To meet the challenge, researchers have proposed a number of different iterative statistical methods to reconstruct the PET images based on different statistical assumptions of PET measurements, such as maximum likelihood (ML), expectation maximization (EM) [3–5], maximum a posteriori (MAP) [6–8], and penalized weighted least-squares (PWLS) [9, 10]. Nevertheless, these iterative statistical methods still have several drawbacks. For example they cannot easily handle low signal-to-noise ratio (SNR) data. Furthermore, after the subtraction of random events, the corrections for scanner sensitivity and dead time, and the corrections for attenuation and scatter, the actual statistical property of measured data is quite complex and it does not exactly follow the Poisson distribution [11, 12].

The nature of PET images is an additional factor to consider in PET image reconstruction [13]. One important PET image feature is the edge. The edge information usually represents the sharp variation in images, such as the object boundaries, which is very important and useful for clinical diagnosis, e.g., of tumors [14]. Given the nature of PET images, researchers have proposed total variation (TV) based methods for both the image space and the projection space in PET image reconstruction [15]. TV was incorporated to provide edge-preserving guidance for the reconstruction [15], and it is well known that it suppresses noise effectively while preserving sharp edges [16–18]. Based on the complexity of the statistical property of PET data, there should be several types of TV-constraint PET imaging models for different statistical assumptions. However, there have been no discussions or research studies on this point in the PET imaging community. In contrast, two types of TV incorporated models have been developed in the image processing community: Gaussian-TV [19] and Poisson-TV [20]. The Gaussian-TV model includes an energy functional to control the type of smoothness of solutions. The Poisson-TV model includes a Poisson data fidelity term to meet the statistical assumption. Both can be used for PET image reconstruction theoretically. However, it remains unknown which one is the better choice for various PET application areas. For example, the low bias and variance of the reconstructed image is more important for kinetic estimation, whereas high contrast is required for tumor diagnosis. In addition, a complication of the TV model is the strong nonlinearity in the data fidelity, and therefore, problems or issues arise in the computation of minimizers [20].

There are three classes of existing solvers for the TV problem. Algorithms in the first class are based on smoothing the TV term, since TV is non-smooth, which causes the main difficulty in solving the TV problem. A number of methods based on smoothing have been proposed, one of which is the second-order cone program (SOCP) [21, 22]. The SOCP solver reformulates TV minimization as a second order cone programming problem [22], which can be solved by interior-point algorithms. The SOCP solver can easily be adapted to various convex TV models with distinct terms and constraints with high accuracy. However, the speed of SOCP is very slow, because it embeds the interior-point algorithm and directly solves a linear system at each iteration. The second class of algorithms for TV problems comprises those based on the iterative shrinkage/thresholding (IST) algorithms, which have been proposed by several researchers in the last few years [23–26]. IST is able to minimize the TV constraint term with some non-quadratic and non-smooth regularization terms. The convergence rate of IST algorithms heavily relies on the linear observation operator, and the convergence rate of algorithms in this class is not sufficiently fast. Furthermore, IST-based algorithms for TV de-convolution require that a de-noising subproblem be solved at each iteration and they cannot take advantage of problem structures. The third class of algorithms for TV problems comprises those based on seeking the minimizer or maximizer of the original constrained problem by a sequence of unconstrained subproblems. Methods belonging to this class add a quadratic penalty term instead of the normal constraint term in the objective function. The penalty term is the square of the constraint violation with the multiplier. Because of its simplicity and intuitive appeal, this approach is widely used. The well-known augmented Lagrangian method (ALM) [27, 28] belongs to this class. In the augmented Lagrangian method, the Lagrangian multiplier is introduced and is estimated at each iteration in the objective function. However, it requires that multipliers go to infinity to guarantee the convergence, which may cause the ill-condition problem numerically.

To overcome the issue that the solvers mentioned above are weak in terms of efficiency and robustness, in this study an alternating direction method (ADM) was applied [29, 30] to solve the TV problem. The proposed ADM constitutes implementable variants of the classical ALM for optimization problems with separable structures and linear constraints. In this method, the TV regularization term is split into two terms with the aid of a new slack variable so that an alternating minimization scheme can be coupled to minimize the approximate objective function. This split and the use of the alternating minimization scheme not only accelerate the convergence rate of the solution, but also result in improved accuracy, as well as in robustness of the reconstructed results to the noise in the data set.

We used Monte Carlo simulated data, phantom data, and real patient data to validate the performance of the proposed algorithm as perceived both quantitatively and visually. Experimental results show that the proposed algorithm is highly effective in preserving sharp image edges and more details while eliminating staircase artifacts. In addition, the performances of the Poisson-TV and LS-TV methods on PET data at different counting levels are also evaluated in this work.

The rest of this paper is organized as follows. In Section 2, first the PET imaging model is reviewed, and then, two objective functions and the corresponding method to solve them are suggested. In Section 3, the experimental setup, results, and comparisons of the existing methods and the proposed methods are presented. Section 4 constitutes the conclusion.

## Materials and Methods

### PET imaging model

PET acquired data are organized in a series of parallel slices that can be reconstructed independently. Every slice of raw data collected by a PET scanner constitutes a list of coincidence events representing near-simultaneous detection of annihilation photons by a pair of detectors. Each coincidence event represents a line in space connecting the two detectors along which the positron emission occurred (the line of response (LOR)). The raw data from PET are organized in a sinogram.

Therefore, PET image reconstruction problems are specific cases of the following general inverse problem: find an estimate of radioactive activity map *u* from a measurement *b* by

$$\begin{array}{c} b=Au+noise \end{array}$$ (1)

In the process of PET imaging reconstruction, *u* is the reconstruction image and *A* is the system matrix that describes the tomographic geometry and the physical factors.

### LS-TV

#### Problem formulation

Assuming piecewise constant behavior of PET images, we introduce total variation (TV) regularization into PET reconstruction.

The problem is formulated as

$$\begin{array}{c} \min_{u}TV\left(u\right),s.t.\;Au=b \end{array}$$ (2)

where *TV*(*u*) is the PET reconstruction with the TV defined regularization; it is defined as ∑_*i*_‖*D*_*i*_*u*‖, the sum of the discrete gradient of activity map *u* of every pixel *i*.

#### Solution

It is difficult to directly obtain the solution of Eq (2). Therefore, we introduce a new auxiliary variable, *w*. At each pixel, an auxiliary variable *w*_*i*_ is introduced into the term ‖.‖. The purpose of this process is to transfer *D*_*i*_*u* out of ‖.‖. The optimization problem of Eq (2) is clearly equivalent to

$$\begin{array}{c} \min_{w_{i},u}\sum_{i}∥w_{i}∥,\;s.t.\;Au=b\;\text{and}\;D_{i}u=w_{i}\;\text{for}\;\text{all}\;i \end{array}$$ (3)

To deal with the constraints, we transform the constrained Problem (3) to an equivalent unconstrained problem using an augmented Lagrangian function [31]. The corresponding augmented Lagrangian function of Problem (3) is

$$\begin{array}{ccc} L_{A}\left(w_{i},u\right) & = & \sum_{i}(∥w_{i}∥-\upsilon_{i}^{T}\left(D_{i}u-w_{i}\right)\\ & & +\;\frac{\beta_{i}}{2}{∥D_{i}u-w_{i}∥}_{2}^{2})-\lambda^{T}\left(Au-b\right)+\frac{\mu }{2}{∥Au-b∥}_{2}^{2} \end{array}$$ (4)

where *υ*_*i*_, *β*_*i*_, *λ*, and *μ* are the multipliers of the four penalty terms. The first term of Eq (4) is the regularization term, and the remaining terms are penalty terms. The second and fourth terms are linear parts, whereas the third term and fifth terms are quadratic parts. These parts ensure the accuracy and robustness of the TV constraint. In order to make the result of every term a number rather than a matrix, transposition of *υ* and *λ* is utilized in Eq (4). To solve Eq (4) efficiently, the ADM, which was originally proposed to handle parabolic and elliptic differential equations, was embedded here. This algorithm is a variant of the classical ALM. When the classical ALM approaches the solutions of the original Problem (4), the nice separable structure emerging from Eq (4) in both the objective function and the constraint is weaker. This drawback, however, can be completely overcome by the ADM. In the ADM, the solution of Eq (4) is transformed to solve three subproblems at each iteration for variables *u*_*k*_ and *w*_*i*,*k*_ and parameters *λ* and *υ*.

In ADM, let *u*_*k*_ and *w*_*i*,*k*_ represent the true minimizers of Eq (4) at the *k*th iteration. *w*_*i*,*k*+1_ can be attained by

$$\begin{array}{ccc} \min_{w_{i}}L_{A}\left(w_{i},u_{k}\right) & = & \sum_{i}(∥w_{i}∥-\upsilon_{i}^{T}\left(D_{i}u_{k}-w_{i}\right)\\ & & +\;\frac{\beta_{i}}{2}{∥D_{i}u_{k}-w_{i}∥}_{2}^{2})-\lambda^{T}\left(Au_{k}-b\right)+\frac{\mu }{2}{∥Au_{k}-b∥}_{2}^{2} \end{array}$$ (5)

To solve this minimization problem, Eq (5) can be separated as two subproblems for variables *u* and *w* based on ADM. We first fix the value of *u* and calculate the solution of *w*. Therefore, only the terms containing *w* are useful. The corresponding subproblem can be expressed as the following problem:

$$\begin{array}{c} w_{i}=\arg\min_{w_{i}}L_{A}\left(w_{i},u_{k}\right)=\min_{w_{i}}\sum_{i}\left(∥w_{i}∥-\upsilon_{i}^{T}\left(D_{i}u-w_{i}\right)+\frac{\beta_{i}}{2}{∥D_{i}u-w_{i}∥}_{2}^{2}\right) \end{array}$$ (6)

For given *β* > 0, the minimizer of Eq (6) is given by the 2D shrinkage-like formula [32]. *w*_*i*,*k*+1_ can be calculated by

$$\begin{array}{c} w_{i,k+1}=\max\{∥D_{i}u_{k}-\frac{\upsilon_{i}}{\beta_{i}}∥-\frac{1}{\beta_{i}},0\}\frac{\left(D_{i}u_{k}-\frac{\upsilon_{i}}{\beta_{i}}\right)}{∥D_{i}u_{k}-\frac{\upsilon_{i}}{\beta_{i}}∥} \end{array}$$ (7)

With *w*_*i*,*k*+1_, we can achieve *u*_*k*+1_ by

$$\begin{array}{ccc} \min_{u}L_{A}\left(w_{i,k+1},u\right) & = & \sum_{i}(∥w_{i,k+1}∥-\upsilon_{i}^{T}\left(D_{i}u_{k}-w_{i,k+1}\right)\\ & & +\;\frac{\beta_{i}}{2}{∥D_{i}u_{k}-w_{i,k+1}∥}_{2}^{2})-\lambda^{T}\left(Au_{k}-b\right)+\frac{\mu }{2}{∥Au_{k}-b∥}_{2}^{2} \end{array}$$ (8)

The constant terms do not influence the minimum, and thus, this subproblem is equivalent to the problem

$$\begin{array}{ccc} u & = & \arg\min_{u}L_{A}\left(w_{i,k+1},u\right)=\min_{u}\sum_{i}(-\upsilon_{i}^{T}\left(D_{i}u_{k}-w_{i,k+1}\right)\\ & & +\;\frac{\beta_{i}}{2}{∥D_{i}u_{k}-w_{i,k+1}∥}_{2}^{2})-\lambda^{T}\left(Au_{k}-b\right)+\frac{\mu }{2}{∥Au_{k}-b∥}_{2}^{2} \end{array}$$ (9)

Its gradient is

$$\begin{array}{c} d_{k}\left(u\right)=\sum_{i}\left(\beta_{i}D_{i}^{T}\left(-D_{i}u-w_{i,k+1}\right)+\mu A^{T}\left(Au-b\right)-A^{T}\lambda -D_{i}^{T}\upsilon_{i}\right) \end{array}$$ (10)

By enforcing *d*_*k*_(*u*) = 0, we can obtain the exact minimizer of Eq (9) directly. However, this calculation is too costly to implement numerically, in particular when the matrix is large. To obtain *u*_*k*+1_ more efficiently, the steepest descent method with an appropriate step length is used iteratively by applying the recurrence formulation:

$$\begin{array}{c} u_{k+1}=u_{k}-\alpha_{k}d_{k}\left(u_{k}\right) \end{array}$$ (11)

where *α*_*k*_ is the step length. Each iteration of the steepest descent method demands that the gradient be updated to update the estimation value of *u*_*k*+1_. Therefore, the step length should be chosen carefully to obtain an accurate solution.

It remains to choose *α*. It is suggested that the aggressive manner [33–35] be used to choose the step length for the steepest descent method, which is called the BB (Barzilai and Borwein) step or BB method. This method is applied to choose *α*:

$$\begin{array}{c} \alpha_{k}=\frac{{\left(u_{k}-u_{k-1}\right)}^{T}\left(u_{k}-u_{k-1}\right)}{{\left(u_{k}-u_{k-1}\right)}^{T}\left(d_{k}\left(u_{k}\right)-d_{k}\left(u_{k-1}\right)\right)} \end{array}$$ (12)

#### Parameters

There are several parameters in the algorithm. Among these, *β*, *υ*_*i*_, and *λ* are initialized as 1, 1, **1**, based on [36]. *μ* is the most important parameter, since it determines the weight of the data fitting term. Therefore, to achieve the best performance, the value of *μ* should be set according to the noise level in observation *b*. For example, the higher the noise level is, the smaller *μ* should be. *μ* was manually tuned in this study. *υ*_*i*_ and *λ* should be updated provided that Eq (4) is minimized at each iteration. According to the formula proposed by Hestenes and Powell [28, 37–40], the update formulas of multipliers follow

$$\begin{array}{c} \upsilon_{i,k+1}=\upsilon_{i,k}-\beta_{i,k}\left(D_{i}u_{k}-w_{i,k}\right) \end{array}$$ (13)

$$\begin{array}{c} \lambda_{k+1}=\lambda_{k}-\mu_{k}\left(Au_{k}-b\right) \end{array}$$ (14)

The program terminates after a certain number of iterations (300 in this study), or when the relative stopping criterion (based on empirical estimations) is reached:

$$\begin{array}{c} \epsilon =\frac{∥u^{k+1}-u^{k}∥}{∥u^{k}∥}<10^{-3} \end{array}$$ (15)

### Poisson-TV

#### Problem formulation

Because of the issue of the low SNR and the Poisson distribution of PET measurements, we build the objective function as

$$\begin{array}{c} \min_{u}TV\left(u\right)+\mu \sum_{i}\left(\bar{b_{i}}-b_{i}\log\bar{b_{i}}\right),s.t.\;Au=b \end{array}$$ (16)

#### Solution

To solve the objective function in Eq (16), the ADM is also used to solve some subproblems at each iteration to approach a solution of Eq (16). We add two auxiliary variables, *Z*_*S*_ and *S*, into the function, as in Eq (3). Then, we construct the augmented Lagrangian objective function for Eq (16):

$$\begin{array}{c} \min_{u}TV\left(u\right)+\mu \sum_{i}\left(\bar{b_{i}}-b_{i}\log\bar{b_{i}}\right)-<Z_{S},S-u>+\frac{\beta_{S}}{2}{∥S-u∥}_{F}^{2} \end{array}$$ (17)

In a more compact form, we have

$$\begin{array}{c} \min_{u}\sum_{i}∥Du_{i}∥+\mu \sum_{i}\left(\bar{b_{i}}-b_{i}\log\bar{b_{i}}\right)+\frac{\beta_{S}}{2}{∥S-u-\frac{Z_{S}}{\beta_{S}}∥}_{F}^{2} \end{array}$$ (18)

We can obtain *u* by

$$\begin{array}{c} \min_{u}\sum_{i}∥Du_{i}∥+\frac{\beta_{S}}{2}{∥S-u-\frac{Z_{S}}{\beta_{S}}∥}_{F}^{2} \end{array}$$ (19)

where the *u* sub-problem is solved by an EM-like method. We rewrite the objective function with items containing only *u* and take the expectation step with respect to the unobservable variables *w*_*i*_*j* and calculate the surrogate function.

$$\begin{array}{c} F\left(u\right)=\mu \sum_{m=1}^{n_{m}}\left(\sum_{j=1}^{n_{j}}\left[\sum_{i=1}^{n_{i}}\left(u_{jm}aij-w_{ij}^{m}log\left(g_{ij}S_{jm}\right)\right)\right]\right) \end{array}$$ (20)

We minimize this surrogate function *F* by zeroing its derivative with respect to *S*_*jm*_:

$$\begin{array}{c} \frac{\partial F}{\partial S_{jm}}=\mu \sum_{m=1}^{n_{i}}g_{ij}-\frac{\mu }{S_{jm}}\sum_{i=1}^{n_{i}}w_{ij}^{m}=0 \end{array}$$ (21)

Thus, $x_{jm}^{k+1}$ is the root of

$$\begin{array}{c} S_{jm}^{2}+\left(\mu \sum_{i=1}^{n_{i}}g_{ij}\right)x_{jm}-\mu \sum_{i=1}^{n_{i}}w_{ij}^{m}=0 \end{array}$$ (22)

Finally, the updating rule for $x_{jm}^{k+1}$ is

$$\begin{array}{c} x_{jm}^{k+1}=\frac{-\left(\mu \sum_{i=1}^{n_{i}}g_{ij}\right)+\sqrt{{\left(\mu \sum_{i=1}^{n_{i}}g_{ij}\right)}^{2}+4\mu \sum_{i=1}^{n_{i}}w_{ij}^{m}}}{2} \end{array}$$ (23)

Moreover, *Z*_*S*_ in Eq (19) is updated as

$$\begin{array}{c} Z_{S}^{k+1}=Z_{S}^{k}-\beta_{S}\left(u^{k+1}-S^{k+1}\right) \end{array}$$ (24)

### Convergence analysis

The analysis of the convergence properties of the proposed TV regulation process is presented in this section. *β* and *β*_*S*_ strongly affect convergence in both the Poisson-TV and LS-TV methods. The selection of these parameters also affects the convergence rate of the proposed method. The convergence of the sequence (*w* and *u*) in the Poisson-TV and (*u* and *S*) in the LS-TV was proven in the convergence analysis section in [32, 41]. It has been proven that the proposed method can converge to the final solutions from any initial points. To demonstrate the effectiveness of the ADM, we also provide a comparison of the convergence speed of the proposed method and IST method (a detailed description of this is algorithm is given in [23]) in the results section.

### Ethics Statement

Before the Results section, we state that no human and animal research was involved in this work.

## Results

In this section, to validate and evaluate the proposed method, the results of simulation and clinical experiments are presented. In the Monte Carlo simulation experiments, a Hoffman brain phantom and Zubal phantom were used and we simulated projections using GATE [42]. All the tests were performed using MATLAB on a PC with an Intel i7-3770 3.40 GHz and 8 GB RAM. In the clinical experiment, we applied our method to a typical PET scanner. All the scans were executed using a Hamamatsu PET scanner. To obtain a good visual effect of our method, we compared the EM [43], Poisson-TV, and LS-TV methods.

### Monte Carlo simulations

**Fig 1:**
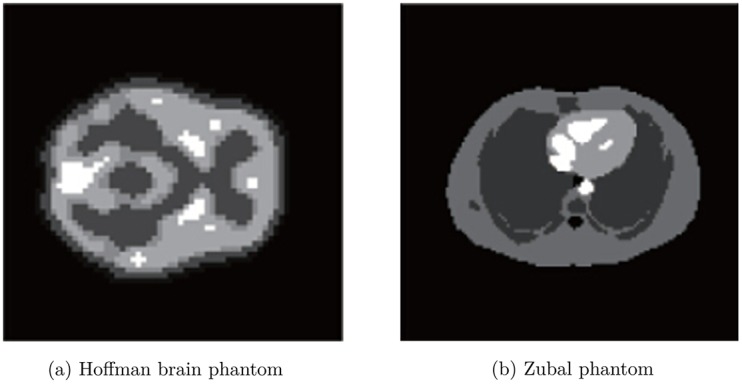
Ground truths of the Monte Carlo simulation.

The scanner simulated in GATE was the Hamamatsu SHR-22000 scanner, which consists of 32 rings with 24576 (3072/ring) BGO crystals of 2.8*mm* ∗ 6.95*mm* ∗ 30*mm*, 768 PMTs, and an 838 mm detector ring diameter. The activity maps of the Hoffman brain phantom (Fig 1(a)) and Zubal phantom (Fig 1(b)) were used as the ground truths. The sinograms calculated from the ground truths by GATE were used as the measurements for our tests. All the tests were performed using MATLAB on a PC with an Intel i7-3770 3.40 GHz and 8 GB RAM. The system matrix *G* used for matching the dimensions of the simulated sinogram was generated by the Fessler tomography toolbox [44].

Both the EM and our model-based TV method were applied to reconstruct the activity maps from the Monte Carlo simulation measurements. The ability of two frameworks (the EM and model-based TV method) to reconstruct a PET activity map was compared in this experiment. This ability is quantified using the relative errors bias, variance, and contrast recovery coefficient (CRC) of the region of interest (ROI) calculated as

$$\begin{array}{c} bias=\frac{1}{n}\sum_{i=1}^{n}\left({\hat{u}}_{i}-u_{i}\right) \end{array}$$ (25)

$$\begin{array}{c} variance=\frac{1}{n}\sum_{i=1}^{n}{\left({\hat{u}}_{i}-u_{i}\right)}^{2} \end{array}$$ (26)

$$\begin{array}{c} CRC=\frac{Contrast_{measure}}{Contrast_{theory}}=\frac{{\left(S/B\right)}_{measure}-1}{{\left(S/B\right)}_{theory}-1} \end{array}$$ (27)

where *u*_*i*_ is the *i*th pixel of ground truth *u* and ${\hat{u}}_{i}$ is the *i*th pixel of the reconstructed images. *S* is the mean activity of the ROI and *B* is the mean activity of the white matter region (background) in the reconstructed image. The bias and variance are used to evaluate the accuracy of the reconstruction and CRC is used to indicate the relative contrast of the ROI in the reconstructed images.

**Fig 2:**
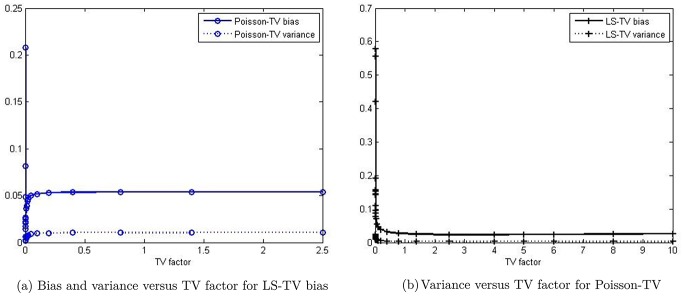
Profile of the bias and variance versus TV factor.

In our method, the TV factor (the value of weighting parameter *μ*) is an important parameter, which can be optimized to smoothen the results. To study the impact of the initial condition of the TV factor in our method, we plotted the bias curve and variance curve versus the TV factor, which are shown in Fig 2. When the TV factor increases, the bias and variance curves first decrease and then increase. Therefore, we can choose the nadir value of the curve as the optimized TV factor, which is 0.0025 in Poisson-TV and 4 in LS-TV for all experiments at all count levels.

**Fig 3:**
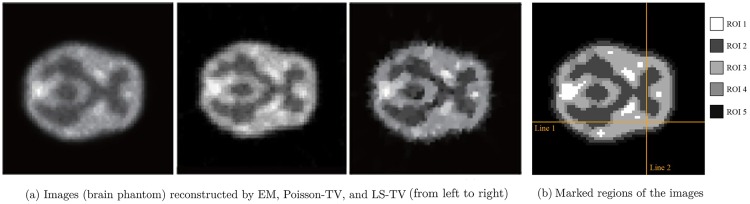
Reconstruction of the brain phantom.

**Fig 4:**
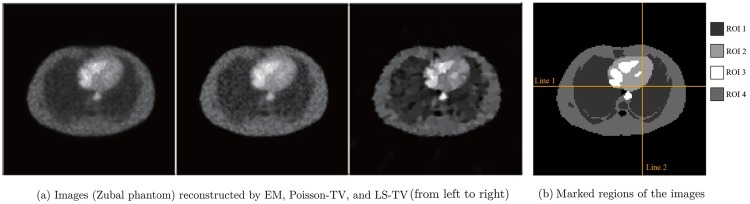
Reconstruction of the Zubal phantom.

**Fig 5:**
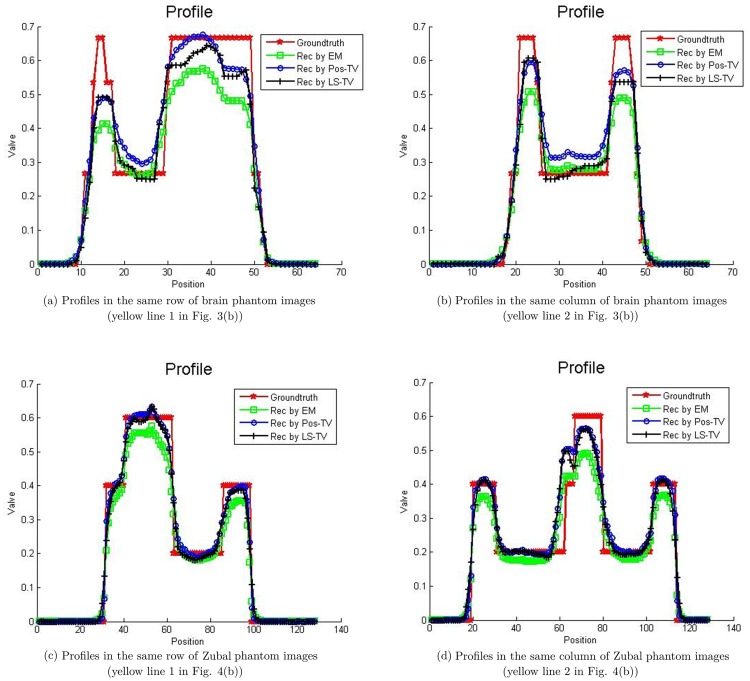
Profiles of the marked region of the brain phantom.

The activity maps of Hoffman brain phantom and Zubal phantom reconstructed by the EM and the proposed TV methods are presented in Figs 3 and 4. The sinograms used in Figs 3 and 4 constitute high count level data; the count is approximately 10^7^. This clearly indicates that the quality of recovery of both Poisson-TV and LS-TV is much higher than that of the EM method. In addition, the reconstructed images also indicate that the edge of the reconstruction produced by the proposed methods is sharper. However, the LS-TV method yields sharper edge information than does Poisson-TV. To evaluate the accuracy of the reconstruction, we marked the specific region of the images (Figs 3(b) and 4(b)) for quantitative analysis. In addition, we present the profiles of the reconstructed images by all methods in the Fig 5. It is clear that the results of Poisson-TV give the closest fit to the ground truth.

To evaluate the performance of the Poisson-TV and LS-TV methods at different count (dose) levels, five counting level values (total number of photon counts in the reconstruction plane: 5 ∗ 10^5^, 1 ∗ 10^6^, 3 ∗ 10^6^, 6 ∗ 10^6^ and 9 ∗ 10^6^) were simulated in this experiment.

**Fig 6:**
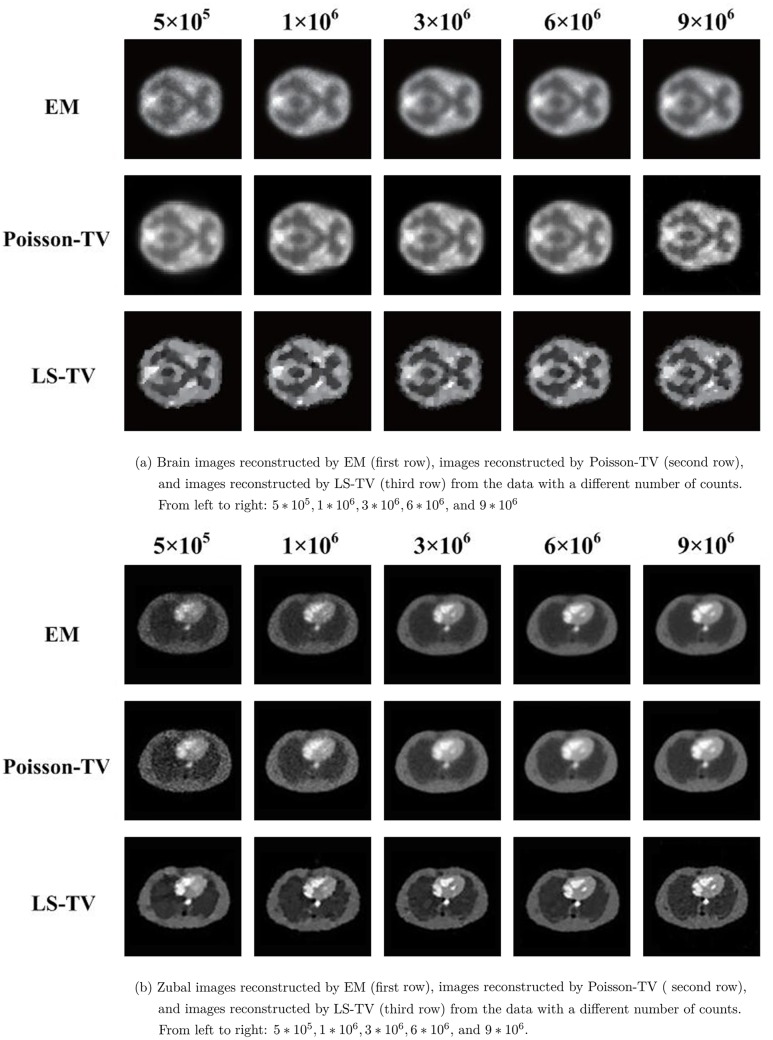
Reconstruction of the EM, LS-TV, and Poisson-TV for the data with different counts.

**Fig 7:**
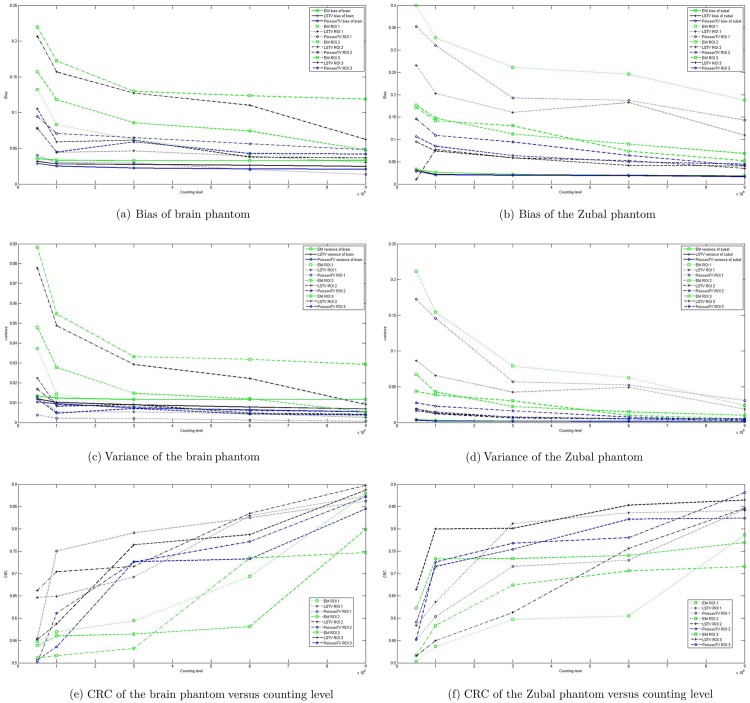
Bias, variance, and CRC profiles for the Zubal phantom and brain phantom with different counts.

The activity maps of the Hoffman brain phantom (Fig 6(a)) and Zubal phantom (Fig 6(b)) reconstructed by EM, Poisson-TV, and LS-TV from the data with different numbers of counts are given in Fig 6. Tables 1 and 2 list the bias and variance of EM, Poisson-TV, and LS-TV for the different counting level data. This demonstrates the statistical analysis of the reconstructed results, which confirms that our model yields better estimates in terms of bias when the counting level changes. The robustness of the proposed method to noise was also evaluated by sinogram data with different counting levels (a lower count means more serious noise). As indicated in Tables 1 and 2, the variance of the Poisson-TV and LS-TV methods is 22% to 74% less than that of the EM method at all counting levels. In other words, the proposed method is more robust to noise than EM. Moreover, Table 1 also shows that the bias and variance for LS-TV and Poisson-TV decrease faster than those of EM when the counting level increases. For a full comparison, the bias, variance, and CRC curves of EM, Poisson-TV, and LS-TV in ROI 1, 2, and 3 for the brain and Zubal phantoms are given in Fig 7. Fig 7(a) and 7(b) show the bias curves. The bias curve for the EM was not improved significantly when the counting level increased, whereas the bias curve for our method decreased. Fig 7(c) and 7(d) show the variance curves, which exhibit the same trend as Fig 7(a) and 7(b). Fig 7(e) and 7(f) show the CRC curves. All the CRC curves increase when the counting level increases, whereas the CRC curves for the proposed method increase faster and higher, and LS-TV gives the best CRC in all methods.

**Table 1:** Quantitative analysis of the reconstructions by EM, Poisson-TV, and LS-TV for Zubal phantom.

	Counting	Zubal Phantom					
		Bias			Variance		
		EM	LSTV	PosTV	EM	LSTV	PosTV
Total	5E5	0.0327	0.0312	**0.0286**	0.0043	0.0039	**0.0033**
	1E6	0.0262	0.0214	**0.0206**	0.0029	0.0021	**0.0020**
	3E6	0.0218	0.0205	**0.0195**	0.0022	**0.0018**	0.0019
	6E6	0.0198	0.0196	**0.0185**	0.0020	0.0017	**0.0017**
	9E6	0.0186	0.0181	**0.0166**	0.0018	**0.0014**	0.0015
ROI 1	5E5	0.3995	**0.2653**	0.3527	0.2114	**0.0864**	0.1725
	1E6	0.3275	**0.2031**	0.3101	0.1541	**0.0657**	0.1455
	3E6	0.2613	**0.1604**	0.1925	0.0789	**0.0425**	0.0571
	6E6	0.2460	**0.1825**	0.1874	0.0626	**0.0492**	0.0523
	9E6	0.1876	**0.1093**	0.1431	0.0236	**0.0186**	0.0309
ROI 2	5E5	0.1715	**0.1005**	0.1459	0.0436	**0.0193**	0.0275
	1E6	0.1415	**0.0777**	0.1088	0.0385	**0.0122**	0.0227
	3E6	0.1307	**0.0586**	0.0943	0.0303	**0.0064**	0.0164
	6E6	0.0735	**0.0524**	0.0642	0.0098	**0.0051**	0.0075
	9E6	0.0521	**0.0348**	0.0412	0.0054	**0.0025**	0.0038
ROI 3	5E5	0.1766	**0.0946**	0.1063	0.0672	**0.0163**	0.0186
	1E6	0.1478	**0.0742**	0.0848	0.0427	**0.0130**	0.0147
	3E6	0.1120	**0.0588**	0.0637	0.0222	0.0077	**0.0073**
	6E6	0.0896	**0.0419**	0.0499	0.0149	0.0054	**0.0051**
	9E6	0.0686	**0.0404**	0.0446	0.0101	0.0052	**0.0048**
ROI 4	5E5	0.1954	**0.1122**	0.1965	0.0865	**0.0186**	0.0822
	1E6	0.1679	**0.0960**	0.1591	0.0595	**0.0186**	0.0539
	3E6	0.1510	**0.0829**	0.1378	0.0432	**0.0155**	0.0366
	6E6	0.1407	**0.0751**	0.1207	0.0387	**0.0131**	0.0281
	9E6	0.0869	**0.0533**	0.0756	0.0174	**0.0070**	0.0132

**Table 2:** Quantitative analysis of the reconstructions by EM, Poisson-TV, and LS-TV for brain phantom.

	Counting	Zubal Phantom					
		Bias			Variance		
		EM	LSTV	PosTV	EM	LSTV	PosTV
Total	5E5	0.0357	0.0319	**0.0273**	0.0134	0.0119	**0.0098**
	1E6	0.0334	0.0281	**0.0249**	0.0123	0.0102	**0.0086**
	3E6	0.0329	0.0275	**0.0223**	0.0116	0.0089	**0.0075**
	6E6	0.0329	0.0263	**0.0201**	0.0116	0.0078	**0.0069**
	9E6	0.033	0.0248	**0.0195**	0.0116	0.0069	**0.0064**
ROI 1	5E5	0.1321	0.0776	**0.0400**	0.0372	0.0132	**0.0037**
	1E6	0.0834	0.0444	**0.0298**	0.0143	0.0054	**0.0022**
	3E6	0.0620	0.0464	**0.0294**	0.0083	0.0054	**0.0021**
	6E6	0.0423	0.0391	**0.0203**	0.0046	0.0045	**0.0012**
	9E6	0.0337	0.0301	**0.0136**	0.0032	0.0025	**0.0006**
ROI 2	5E5	0.2198	0.2068	**0.0945**	0.0882	0.0779	**0.0168**
	1E6	0.1723	0.1568	**0.0708**	0.0547	0.0488	**0.0090**
	3E6	0.1297	0.1272	**0.0649**	0.0331	0.0292	**0.0079**
	6E6	0.1235	0.1102	**0.0563**	0.0318	0.0222	**0.0060**
	9E6	0.1189	0.0624	**0.0482**	0.0292	0.0091	**0.0054**
ROI 3	5E5	0.1570	0.1051	**0.0780**	0.0479	0.0223	**0.0120**
	1E6	0.1183	0.0591	**0.0448**	0.0277	0.0082	**0.0047**
	3E6	0.0857	0.0615	**0.0591**	0.0147	0.0090	**0.0071**
	6E6	0.0742	**0.0378**	0.0428	0.0120	0.0048	**0.0042**
	9E6	0.0476	**0.0367**	0.0421	0.0053	0.0041	**0.0039**
ROI 4	5E5	0.1542	0.1102	**0.0657**	0.0557	0.0358	**0.0125**
	1E6	0.1199	0.0928	**0.0551**	0.0401	0.0233	**0.0080**
	3E6	0.0932	0.0804	**0.0478**	0.0228	0.0175	**0.0060**
	6E6	0.0758	0.0648	**0.0477**	0.0174	0.0109	**0.0059**
	9E6	0.0716	0.0447	**0.0361**	0.0133	0.0057	**0.0040**
ROI 5	5E5	0.2673	0.2255	**0.1984**	0.2239	0.1677	**0.1469**
	1E6	0.2554	0.2201	**0.1895**	0.2203	0.1626	**0.1414**
	3E6	0.2403	0.2024	**0.1641**	0.2035	0.1356	**0.1156**
	6E6	0.2302	0.1971	**0.1565**	0.1812	0.1317	**0.0909**
	9E6	0.2200	0.1864	**0.1503**	0.1807	0.1239	**0.0899**

**Fig 8:**
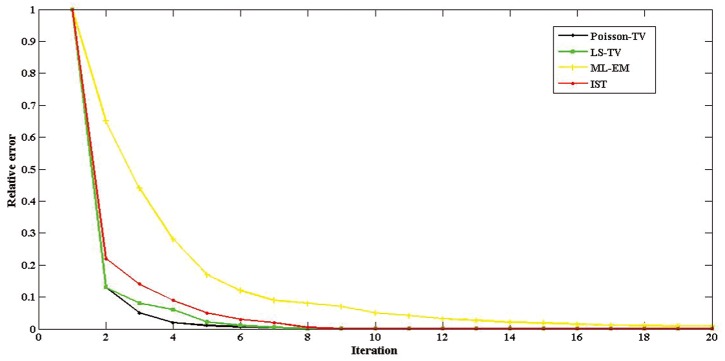
Convergence rate for IST, EM, Poisson-TV, and LS-TV.

Furthermore, we plotted the relative error of reconstruction after each iteration of the algorithms to evaluate the convergence speed of the Poisson-TV, LS-TV, EM, and IST methods. Here, the IST method was used to evaluate the effectiveness of the ADM to solve the TV-problem. The results are shown in Fig 8. The *y*-axis represents relative error and the *x*-axis represents the iteration times of the methods. The black curve represents Poisson-TV, the green curve LS-TV, the yellow curve EM, and the red curve the IST method. For all methods, the relative error approaches close to 0 within 20 iterations (since the stopping threshold is 10^−3^ for all experiments). With the help of the ADM, the relative error of the Poisson-TV and LS-TV decreases to approximately 0.1 with only two iterations, while the relative error of IST decreases to approximately 0.22 and the relative error of EM is 0.65. This shows that the curves of both Poisson-TV and LS-TV decrease faster than those of IST and EM. All the results demonstrate that the convergence speed of the ADM is faster than that of the IST method and much faster than that of the EM method.

### Phantom case

**Fig 9:**
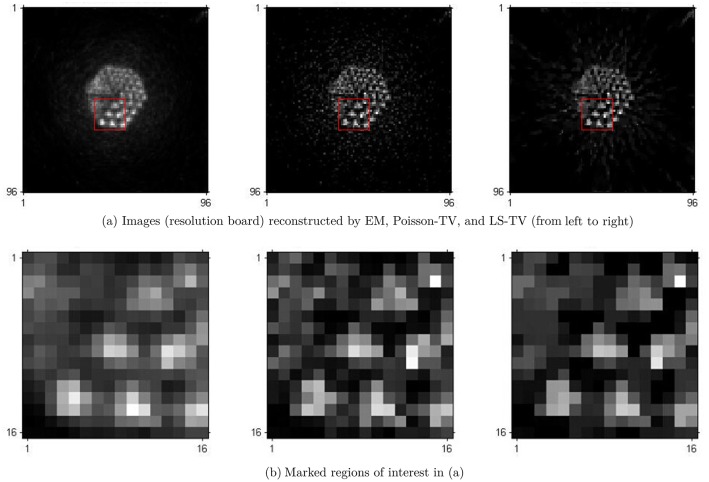
Reconstruction of the phantom case.

In this section, the results of real scanning phantom experiments are presented. The results were obtained under real conditions, in which sinograms were generated by the scan of a Hamamatsu SHR-22000 PET scanner. The phantom used is the Derenzo phantom. There are six sector regions in this phantom. In each sector region, there are several spheres arranged in a triangular array. These spheres have different diameters corresponding to the different spatial resolutions. A ^18^*F* − *FDG* solution was injected into the Derenzo phantom. The total counting rate of coincidence events was 10^5^. The images reconstructed by the EM, Poisson-TV, and LS-TV methods are shown in Fig 9. To evaluate the detailed information of the reconstruction, the marked areas (marked by a red rectangle) are given in Fig 9(b) and zoomed for comparison. It is clearly indicated that the quality of the recovery, in particular the detailed information yielded by the Poisson-TV and LS-TV methods, is better than that of the EM method.

### Clinical case

In this section, we validate the proposed method using clinical patient data. The clinical patient data comprised a PET scan acquired from a volunteer at a local hospital. The PET system used was a Hamamatsu SHR-22000 whole body PET scanner. It has 32 crystal rings and can be operated in 2D or 3D mode. The trans-axial resolution of the central FOV is about 3.7 mm. The patient lay on the bed and the main target of the scanning was the thorax. The total scanning time was 40 min. Random, normalization, dead time, scatter, and attenuation correction were applied to the measurement data using the programs provided by the scanner prior to reconstruction. The measurement data were stored in the sinogram model, the size of the reconstructed images was 128 ∗ 128.

**Fig 10:**
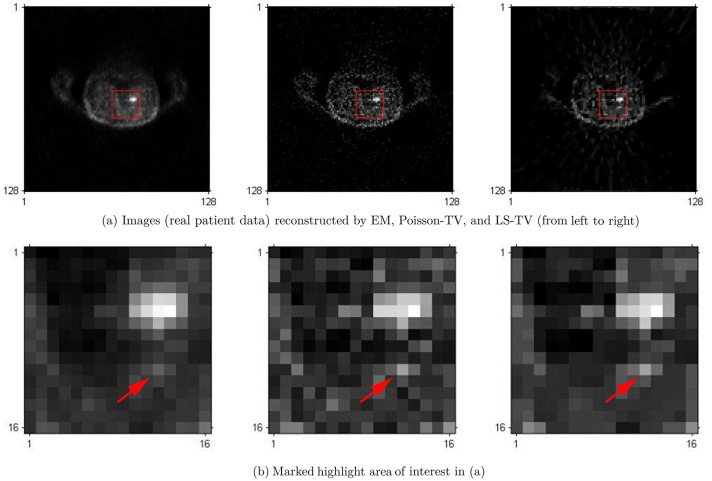
Reconstruction of images for the clinical patient.

To evaluate the effectiveness of the proposed method, the Poisson-TV, LS-TV, and EM algorithms were used in this experiment. Their results are shown in Fig 10. In Fig 10(a), the difference between these three methods is not sufficiently clear, however, all the algorithms can provide a clear reconstruction. However, when we zoom in on the highlighted region (marked by a red rectangle) in the reconstructed images, it is clear that, as compared to EM, both the Poisson and LS TV algorithm can provide better edge information and better visual quality, in particular for the small highlighted point marked by the red arrow. In the results of the EM algorithm, this point almost disappears in the noise background. In contrast, we can easily locate this point in the results of Poisson-TV and LS-TV. In the comparison of the LS-TV and Poisson-TV, since the results of the LS-TV method suffer from some undesirable artifacts (such as edge over-smoothness and staircase effects), Poisson-TV yields a much better visual quality than LS-TV and recovers more detail of textures in the reconstructed image.

## Conclusion

This paper presented a TV-constraint reconstruction algorithm for low dose PET image reconstruction. According to the property of the PET measured data, two types of TV-constraint models, LS-TV and Poisson-TV, were established. To efficiently obtain high quality reconstructed images, the ADM is used. To evaluate the effectiveness of the proposed LS-TV and Poisson-TV methods, PET data measured from a Monte Carlo simulation and real scanning were used. As compared with the traditional EM method, the proposed TV-constraint models are able to reconstruct the images with higher accuracy and much clearer structures. The ADM also provides a faster convergence rate as compared to the IST method for solving the TV problem. Both Poisson-TV and LS-TV can provide a high visual quality and are robust to noise corruptions at a low dose level; Poisson-TV gives a more accurate estimation with lower bias and variances, whereas the CRC of the LS-TV is better. This means that, although both the proposed TV methods can provide a highly accurate and robust reconstruction, Poisson-TV is more suitable for kinetic parameter estimation, which requires a highly accurate estimation with low bias and variance for PET images. In contrast, LS-TV is more suitable for tumor diagnosis, which requires a high contrast for PET images in order to locate the tumor.

## Abbreviations

TV: Total variation
ADM: Alternating direction method
EM: Expectation-maximization
Poisson-TV: Poisson total variation
LS-TV: Least square total variation
MAP: Maximum a posteriori
PWLS: Penalized weighted lease-squares
SNR: Signal-to-noise ratio
SOCP: Second-Order cone program
LOR: Line of response
BB: Barzilai and Borwein
CRC: Contrast recovery coefficient
ROI: Region of interest
FOV: Field of view
